# Impending Eclampsia With Protein C Deficiency in Pregnancy: A Case Report From Recovery to Mortality

**DOI:** 10.7759/cureus.64289

**Published:** 2024-07-10

**Authors:** Sai Kalmegh, Meenal Patvekar

**Affiliations:** 1 Obstetrics and Gynecology, Dr. D. Y. Patil Medical College, Hospital & Research Centre, Pune, Pune, IND

**Keywords:** impending eclampsia, thrombophilia, hypercoagulable state, preeclampsia, protein c deficiency

## Abstract

This case report details a 30-year-old pregnant woman with inherited protein C deficiency who developed severe preeclampsia (PE), intrauterine growth restriction, and pancytopenia. Despite treatment, including anticoagulants, her condition worsened, resulting in postpartum mortality. Protein C deficiency, integral to the coagulation cascade, can exacerbate pregnancy’s hypercoagulable state, contributing to adverse outcomes like PE. Mutations in the protein C gene can cause type I or type II deficiency, affecting protein C antigen and activity levels. The patient’s history of recurrent pregnancy losses and conception via in vitro fertilization despite advisories highlights the complexities of managing pregnancy complications with protein C deficiency. Although some studies do not establish a direct link between protein C levels and PE, further research should explore thrombophilia’s role in PE development and recurrence. Prospective studies investigating antithrombotic strategies in thrombophilic pregnancies could offer insights into reducing PE recurrence risks. This case underscores the need for multidisciplinary management and ongoing research to enhance clinical strategies and outcomes in high-risk pregnancies involving protein C deficiency.

## Introduction

Protein C is a glycoprotein that is mostly produced in the liver and makes up the bulk of the body’s natural anticoagulant system. It is vital for preserving physiologic hemostasis and is dependent on vitamin K [1,2]. The hypercoagulable state of pregnancy favors venous stasis and thromboplastin release. The presence of protein C helps to maintain the equilibrium of the anticoagulant system and thereby counterbalances pregnancy hypercoagulability. This results in unchecked thrombin generation and thromboembolism. In rare events where pregnancy coexists with protein C deficiency, the outcome worsens in the form of recurrent pregnancy losses, intrauterine growth restriction, and stillbirth. One such known complication is preeclampsia (PE). Thrombophilias such as protein C deficiency are risk factors in the development and progression of PE [3]. PE’s origin is still unknown, although theories include vascular endothelium destruction, intervillous and spiral artery thrombosis, and irregularities in coagulation, which result in insufficient circulation in the mother, fetus, and placenta [4,5]. Thus, PE, along with an underlying protein C deficiency, causes deterioration of the coagulation cascade and hampers hemodynamic stability, thereby worsening the prognosis. Managing such patients requires a multidisciplinary approach. Here we present a patient with inherited protein C deficiency with impending eclampsia with in vitro fertilization (IVF) conception.

## Case presentation

A 30-year-old female presented to our labor room at 32 weeks gestation with complaints of breathlessness, headache, grade II bilateral pedal edema, and facial puffiness for two days. She had been diagnosed with preeclampsia (PE) at a private hospital four days prior to admission. Her obstetric history included three previous spontaneous losses at one to two months of gestation. Four years ago, she made multiple visits to a hematology center with complaints of epistaxis, hemoptysis, maxillary rash, fatigue, palpitations, and facial puffiness. This pregnancy was an IVF conception. Further evaluation revealed low protein C levels and deficiencies in vitamin B12 and iron. She responded well to B12 supplements and ferric carboxymaltose injections.

Vitals showed an oxygen saturation of 80% in room air and a blood pressure of 150/100 mmHg. Auscultation revealed bilateral crepitations. The abdominal examination indicated a relaxed uterus corresponding to 28 weeks, with fetal heart sounds at 136 bpm. Laboratory investigations (detailed in Table 1) showed a hemoglobin level of 8.1 gm/dl, a platelet count of 67,000/µL, and a prothrombin time/international normalized ratio of 1.29. Urine analysis showed trace proteins. Other tests, including antinuclear antibody blot, arterial blood gas analysis, and anti-B-type natriuretic peptide, were normal, while rheumatoid factor and anti-cyclic citrullinated peptide antibodies were negative. A diagnosis of gravida 4, abortion 3 (G4A3) at 31+2 weeks, with severe preeclampsia, intrauterine growth retardation, pancytopenia, and protein C deficiency, was made. Serum procalcitonin increased to 23.34 over five days. Considering the low counts, a bone marrow biopsy was performed, revealing a dimorphic anemia-like picture. The patient was transferred to the intensive care unit for further observation and management.

**Table 1:** Laboratory investigation profile ABG, arterial blood gas analysis; VBG, venous blood gas analysis

Investigation	Day 1	Day 2	Day 3	Day 4	Day 5	Day 6	Day 7	Day 8	Normal range
Hemoglobin (g/dl)	8.1	8.2	8.1	7.7	-	5.8	5.7	7.1	11.6-15.0
White blood cells (/µL)	9,000	6,800	8,200	4,167	-	7,600	9,600	400	4,000-10,000
Platelets (/µL)	67,000	53,000	61,000	55,000	-	25,000	39,000	12,000	150,000-410,000
Total bilirubin (mg/dl)	1.5	-	-	-	-	2.8	2.2	6.4	0.22-1.20
Bilirubin conjugated (mg/dl)	0.7	-	-	-	-	0.9	0.9	2.3	Up to 0.5
Bilirubin unconjugated (mg/dl)	0.8	-	-	-	-	1.9	1.3	4.1	0.1-1.0
Alkaline phosphatase (U/Lt)	399	-	-	-	-	50	32	30	35-104
Random blood sugar (mg/dl)	78	-	-	-	-	-	-	-	Less than 200
Total protein (g/dl)	5	-	-	-	4.8	-	-	2.3	6.4-8.3
Albumin (g/dl)	2.2	-	-	-	2.5	-	-	1.3	3.5-5.2
Globulin (g/dl)	2.8	-	-	-	2.3	-	-	1	2.3-3.5
Lactate dehydrogenase (U/Lt)	349	-	-	-	-	419	-	358	81-234
Uric acid (mg/dl)	6.8	-	-	-	-	-	-	7	2.7-6.1
Sodium (mmol/Lt)	130	134	136	135	136	137	139	137	136-145
Potassium (mmol/Lt)	5.4	6.1	6	7	5.5	5.5	4	3.7	3.5-5.1
Chloride (mmol/Lt)	108	111	115	118	117	110	112	109	98-107
Urea (mg/dl)	51	-	-	-	-	93	73	73	17-49
Creatinine (mg/dl)	0.81	-	-	-	-	0.95	0.83	1.07	0.6-1.2
Thyroid-stimulating hormone (𝜇IU/ml)	1.99	-	-	-	-	-	-	-	0.3-3.0
Activated partial thromboplastin time (s)	26	-	-	24.5	-	-	-	85	2.24-26.78
Prothrombin time/international normalized ratio (s)	13.50/1.24	-	14.1/1.29	14/1.28	-	15.5/1.42	-	44.2/4	8.97-12.91/0.85-1.15
D-Dimer (ng/ml)	1,540	-	-	1,548	-	1,002	-	3,450	0-500
Fibrinogen (mg/dl)	146.9	-	-	181.5	169.6	185.4	-	115.5	238-498
ABG									
pH	7.35	7.43	-	7.43	7.4	-	-	7.4	7.35-7.45
pCO2 (mm Hg)	27	25	-	35	32.4	-	-	32.4	35-45
pO2 (mm Hg)	71	77	-	165	148	-	-	126	83-108
Bicarbonate (HCO3) (meq/Lt)	14.9	16.6	-	23.2	19.8	-	-	11.9	21-28
VBG									
pH	-	7.47	-	7.45	-	-	7.43	-	7.32-7.42
pCO2 (mm Hg)	-	25	-	28	-	-	38	-	41-51
pO2 (mm Hg)	-	77	-	85	-	-	27	-	25-40
Bicarbonate (HCO3) (meq/Lt)	-	18.2	-	19.5	-	-	25.2	-	21-28

The patient received an IV labetalol infusion (20 mg over 10 minutes), IV magnesium sulfate (4 gm) with an additional 5 gm intramuscularly, aspirin 150 mg daily, labetalol 100 mg thrice daily, and IV dexamethasone (12 mg twice daily per Prichart’s regimen). Following stabilization of blood pressure and improvement in platelet count via fresh frozen plasma (FFP) transfusion, an elective cesarean section was performed due to severe preeclampsia, intrauterine growth restriction, and pancytopenia with protein C deficiency. Postpartum, the patient received transfusions: 1 unit PRBC and 4 units FFP on day 1, followed by 2 units PRBC, 2 single donor platelets, and 4 units FFP over the next three days. On day 4, vitals included a BP of 80/40 mm Hg and oxygen saturation of 80%. Subsequently, the patient developed tachypnea and tachycardia, necessitating ionotropic support and intubation. Despite efforts, the patient had a flatlined ECG and passed away. The cause of death was disseminated intravascular coagulopathy with protein C deficiency and impending eclampsia, complicated by neutropenic sepsis.

## Discussion

Activated protein C plays a critical role in the coagulation cascade by neutralizing factors Va and VIIIa. Protein C deficiency, although uncommon, is significant in pregnancy due to its association with hypercoagulability [2,6]. This deficiency can be inherited or acquired, with inherited cases typically manifesting as an autosomal recessive disorder caused by mutations in the protein C gene (PROC) on chromosome 2q14.3. More than 160 mutations in PROC have been documented and categorized into two main types: type I, characterized by low levels of both protein C antigen and activity [7,8], and type II, characterized by normal antigen levels but reduced activity of protein C.

In our case, the patient was counseled against conception owing to her bad obstetric history. In spite of that, she conceived through IVF. The patient was on anticoagulants throughout the pregnancy, yet the underlying deficiency manifested in the form of severe PE and further exacerbated it. Despite numerous transfusions, the patient’s condition deteriorated, and she could not be revived.

A similar study was reported by Okoye et al. [8], wherein a cross-sectional study was conducted among 90 pregnant women with PE. Plasma levels of protein C and protein S were analyzed. However, they found no association between PE and protein C and protein S levels. Anant et al. reported a case of protein C deficiency with a successful outcome in twin pregnancy in a woman with a bad obstetric history and a history of thrombosis [9].

To determine whether hereditary or acquired thrombophilia raises the risk of PE development and recurrence, more prospective trials are required. In this context, a small number of observational studies indicate that preventive low-molecular-weight heparin doses from the start of pregnancy may lower thrombophilic women’s PE recurrence rate [1,2]. These findings emphasize the necessity of conducting randomized trials of antithrombotic intervention to stop PE from recurring in thrombophilic women and to determine whether or not such an intervention is cost-effective.

## Conclusions

This case report underscores the complexities of managing impending eclampsia, particularly in patients with underlying thrombophilia like protein C deficiency. The unpredictable course observed, with initial improvement followed by rapid deterioration, necessitates a high index of suspicion for impending eclampsia in this high-risk population. Early recognition and aggressive management strategies are paramount to optimize maternal and fetal outcomes.

Moving forward, further research is crucial to uncover the pathophysiological mechanisms linking protein C deficiency to impending eclampsia. Exploring innovative therapeutic approaches offers potential for reducing complication rates and enhancing maternal-fetal health. By advancing our knowledge and management of impending eclampsia, we aim to safeguard the lives of pregnant women and their unborn children.
